# Radionuclide-Based Cancer Imaging Targeting the Carcinoembryonic Antigen

**DOI:** 10.4137/bmi.s1124

**Published:** 2008-09-23

**Authors:** Hao Hong, Jiangtao Sun, Weibo Cai

**Affiliations:** 1 Departments of Radiology and Medical Physics, School of Medicine and Public Health, University of Wisconsin—Madison, Madison, Wisconsin, U.S.A; 2 University of Wisconsin Paul P. Carbone Comprehensive Cancer Center, Madison, Wisconsin, U.S.A

**Keywords:** carcinoembryonic antigen, single photon emission computed tomography (SPECT), positron emission tomography (PET), antibody, antibody fragment, pretargeting

## Abstract

Carcinoembryonic antigen (CEA), highly expressed in many cancer types, is an important target for cancer diagnosis and therapy. Radionuclide-based imaging techniques (gamma camera, single photon emission computed tomography [SPECT] and positron emission tomography [PET]) have been extensively explored for CEA-targeted cancer imaging both preclinically and clinically. Briefly, these studies can be divided into three major categories: antibody-based, antibody fragment-based and pretargeted imaging. Radiolabeled anti-CEA antibodies, reported the earliest among the three categories, typically gave suboptimal tumor contrast due to the prolonged circulation life time of intact antibodies. Subsequently, a number of engineered anti-CEA antibody fragments (e.g. Fab’, scFv, minibody, diabody and scFv-Fc) have been labeled with a variety of radioisotopes for CEA imaging, many of which have entered clinical investigation. CEA-Scan (a ^99m^Tc-labeled anti-CEA Fab’ fragment) has already been approved by the United States Food and Drug Administration for cancer imaging. Meanwhile, pretargeting strategies have also been developed for CEA imaging which can give much better tumor contrast than the other two methods, if the system is designed properly. In this review article, we will summarize the current state-of-the-art of radionuclide-based cancer imaging targeting CEA. Generally, isotopes with short half-lives (e.g. ^18^F and ^99m^Tc) are more suitable for labeling small engineered antibody fragments while the isotopes with longer half-lives (e.g. ^123^I and ^111^In) are needed for antibody labeling to match its relatively long circulation half-life. With further improvement in tumor targeting efficacy and radiolabeling strategies, novel CEA-targeted agents may play an important role in cancer patient management, paving the way to “personalized medicine”.

## Introduction

Carcinoembryonic antigen (CEA), a complex and highly glycosylated macromolecule, contains approximately 50% carbohydrate with a molecular weight of around 200 kDa. Normally expressed during the development of the fetal gut, it is also a well-established tumor-associated antigen highly expressed in colorectal carcinoma and frequently elevated in adenocarcinomas of the lung, breast, other gastrointestinal organs and the ovaries (Goldstein and Mitchell, 2005; Schneider, 2006; Ugrinska et al. 2002). In 1981, the National Institutes of Health (NIH) announced that monitoring CEA expression was the best available non-invasive technique for the detection of recurrences in patients with a history of colorectal cancer (1981). Subsequently, CEA measurement has been widely used in the follow-up of patients after resection of colorectal cancer. Because its expression level in normal tissues is quite low, CEA is also a suitable target for cancer intervention (Goldenberg et al. 1978).

Molecular imaging refers to the characterization and measurement of biological processes at the molecular level (Mankoff, 2007; Massoud and Gambhir, 2003). It takes advantage of traditional diagnostic imaging techniques and introduces molecular probes to measure the expression of indicative molecular markers at different stages of diseases. The most frequently used molecular imaging modalities include optical bioluminescence, optical fluorescence, targeted ultrasound, molecular magnetic resonance imaging, single photon emission computed tomography (SPECT) and positron emission tomography (PET). Recently, optical imaging has been employed for intra-operative or non-invasive imaging of CEA-positive tumors (Kaushal et al. 2008; Venisnik et al. 2007). Comparing to optical imaging, radionuclide-based imaging techniques are more advantageous in that they are very sensitive and quantitative with no tissue penetration limit, hence more suitable for clinical translation. Clinically, SPECT and PET has been widely used over the last several decades in cancer patient management, including diagnosis, staging and treatment monitoring (Kelloff et al. 2005). In this review, we will summarize the current state-of-the-art radionuclide-based cancer imaging targeting CEA.

## SPECT and Gamma Camera Imaging

SPECT imaging detects gamma rays (Kjaer, 2006; Peremans et al. 2005). A collimator is used to only allow the emitted gamma photon to travel along certain directions to reach the detector, which ensures that the position on the detector accurately represents the source of the gamma ray. The gamma camera can be used in planar imaging to obtain 2-D images, or in SPECT imaging to obtain 3-D images.

Because of the use of lead collimators to define the angle of incidence, SPECT imaging has a very low detection efficiency (<10^−4^ times the emitted number of gamma rays) (Chatziioannou, 2005). Common radioisotopes used for SPECT imaging are ^99m^Tc (t_1/2_: 6.0 h), ^111^In (t_1/2_: 2.8 d), ^123^I (t_1/2_: 13.2 h) and ^131^I (t_1/2_: 8.0 d). SPECT and gamma camera imaging is currently the most frequently used modality for CEA imaging. Based on the targeting ligands used, they can be broadly divided into the following categories: antibody-based, antibody fragment-based and pretargeted imaging.

### Antibody-based imaging

The use of radiolabeled antibodies for tumor imaging and therapy continues to be an active area of research. Immunoscintigraphy can be an adequate diagnostic tool for colorectal cancer, especially in recurrences. CEA imaging using radiolabeled intact antibodies dated back to the early 1980s (Berche et al. 1982). In 1982, a ^131^I-labeled monoclonal antibody (mAb) against CEA was used clinically for detecting gastrointestinal and medullary thyroid cancers. The results were very promising: SPECT imaging detected 16 out of 17 tumor sites (94%) while only 9 out of 21 (43%) tumor sites were identified by rectilinear scintigraphy (Berche et al. 1982). This pioneering report represents a very important first step for antibody-based imaging of CEA expression in the clinic.

Subsequently, SPECT imaging with a number of antibodies against CEA was reported. For example, ^111^In-labeled ZCE-025 (an anti-CEA mAb that does not react with normal granulocyte glycoproteins) was investigated in several studies for the detection of primary, metastatic, or recurrent colorectal carcinomas (Abdel-Nabi et al. 1988; Abdel-Nabi et al. 1991; Kramer et al. 1988; Patt et al. 1988; Patt et al. 1990). Imaging in patients with rising CEA levels successfully detected metastatic colorectal cancer that could not be detected by other methods, therefore changing patient management in many cases. Interestingly, 1 mg of ^111^In-labeled ZCE-025 co-infused with 39 mg of unlabeled antibody achieved much better performance in detecting colorectal cancer, particularly for liver metastases, than ^111^In-labeled ZCE-025 itself (Patt et al. 1988). Although the exact mechanism(s) for this effect is unknown, partial “blocking” caused by the unlabeled antibody may have changed the biodistribution of the radiopharmaceutical.

In these reports, there was no correlation between serum levels of CEA and lesion detectability with mAb-based imaging. For example, the discrepancy between serum CEA levels and tissue CEA expression in breast cancer patients is well known. Although immunohistochemistry shows positive CEA expression in 70%–90% of the cases in breast cancer, the serum CEA levels are often within the normal range (Lind et al. 1991b). In 1991, immunoscintigraphy with a ^111^In-labeled anti-CEA mAb (F023C5i) was performed in 66 patients suspected for primary lung cancer (Griffin et al. 1991). A sensitivity of 90%, specificity of 45% and accuracy of 85% was achieved. SPECT imaging delineated the lesions better than planar gamma camera imaging in each patient, although it did not reveal any new lesions not seen in planar imaging.

Radioiodine and ^111^In has not been widely used for labeling anti-CEA antibodies due to the high cost, limited availability and relatively poor image quality with most gamma cameras (Hansen et al. 1990; Riva et al. 1988). ^111^In is relatively difficult to conjugate to antibodies. Moreover, it usually has considerable accumulation in the reticuloendothelial organs such as the liver, often a major site for tumor metastasis, which significantly limits its potential in cancer imaging.

^99m^Tc is the most widely used isotope for SPECT imaging (Banerjee et al. 2001; Mease and Lambert, 2001). It emits readily detectable 140 keV gamma rays, about the same energy as that used in conventional diagnostic X-ray instrument. The short half-life of ^99m^Tc allows for rapid scanning procedures and at the same time keeping the total radiation exposure to the patient low. Most importantly, it is readily available with a very low cost. ^99m^Tc has been used to label many anti-CEA antibodies (e.g. BW431/26, IMMU-4 and 88BV59) and used in clinical studies for the detection of colorectal, breast and lung cancer (Barzen et al. 1992; Baum et al. 1989; Behr et al. 1995; Fraile et al. 1991; Gonzalez et al. 1991; Kairemo et al. 1993; Lacic et al. 1999; Lind et al. 1989; Lind et al. 1991a; Lind et al. 1991b; Oriuchi et al. 1995; Patt et al. 1994; Serafini et al. 1998; Sirisriro et al. 1996; Stomper et al. 1995; Takenoshita et al. 1995). It was concluded that ^99m^Tc-based immunoSPECT is a suitable method for cancer diagnosis, especially for recurrences. For primary breast cancer, ^99m^Tc-based immunoSPECT has achieved 83% sensitivity and 69% specificity (Lind et al. 1991b). In primary colorectal cancer patients, the lesions can be scintigraphically detected with a sensitivity of 83% and a specificity of 100%. In recurrent colorectal cancer, a sensitivity of 77% and specificity of 88% was reported (Hach et al. 1992). In 1998, a ^99m^Tc-labeled fully humanized mAb 88BV59 was evaluated in a phase III clinical trial to image recurrent, metastatic, or occult colorectal cancer (Serafini et al. 1998). The trial was a success in that it can provide important and accurate information about the presence and location of malignant lesions in patients which could not be detected by computed tomography (CT) scans.

**Figure 1:**
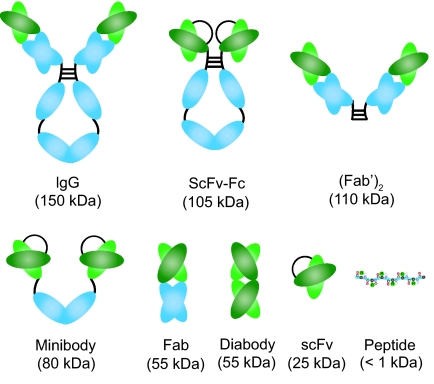
Intact antibodies and a variety of antibody fragments have been explored for CEA-targeted cancer imaging.

Many studies have confirmed that immunoscintigraphy with radio-labeled anti-CEA mAbs is superior to CT for the detection of pelvic and extrahepatic abdominal recurrences of colorectal cancer, while CT is more sensitive in detecting liver and lung metastases. The major reason for this phenomenon is that the blood pool activity typically masks the lung lesion due to the long circulation half-lives of the mAbs and the liver uptake of the mAbs can overshadow the uptake in liver metastases. Besides the limited use in detecting lung and hepatic lesions, the prolonged persistence of the radiolabeled antibody in the circulation can also lead to high background signal. Traditionally, many mAbs are expressed in animals, such as mice, which can induce human anti-mouse antibody (HAMA) activity in many patients (Nussbaum and Roth, 2000; Zbar et al. 2005). Therefore, researchers have explored the use of antibody fragments (Fig. 1) for CEA imaging which may have better tumor targeting and pharmacokinetic properties.

### Fab’ fragment-based imaging

Fab’ fragments of anti-CEA antibodies have been tested for cancer imaging in the early 1990s. Successful imaging of CEA-positive carcinomas within 2 h of intravenous (i.v.) injection of a ^99m^Tc-labeled anti-CEA Fab’ fragment was reported (Goldenberg et al. 1990). Rapid tumor targeting, within a few hours after administration and fast clearance from the blood and normal organs of the Fab’ fragments (blood half-life: 13.2 h) permitted the use of short-lived radionuclides such as ^123^I and ^99m^Tc. Scanning at 2–5 h post-injection (p.i.) of the ^99m^Tc-labeled Fab’ fragment gave a sensitivity of 95% and an accuracy of 94% on a tumor site basis. The radiolabeled Fab’ fragment also revealed a high number of lesions not detectable by other radiological methods, of which 21% were subsequently confirmed as malignant lesions within a 11-month follow-up period. The smallest tumors identified were below 0.5 cm in diameter. This report demonstrated that the ^99m^Tc-labeled Fab’ fragment, which could be prepared by 1-step direct labeling, appears to be the method of choice for rapid and accurate detection of cancer. Although a ^123^I-labeled Fab’ fragment gave slightly better results (sensitivity of 96% and accuracy of 94%) than the ^99m^Tc-labeled version, the limited availability and high cost of ^123^I limited its potential in clinical use.

**Figure 2:**
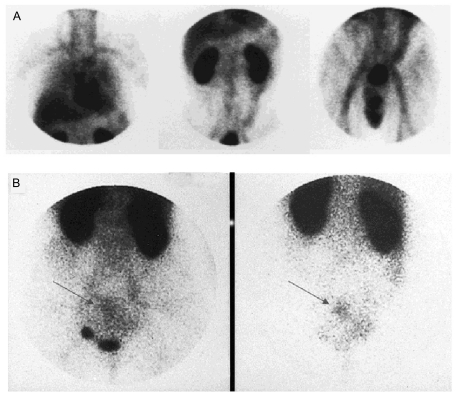
SPECT imaging with CEA-Scan. **A)** Normal biodistribution of CEA-Scan at 4 h post-injection. Anterior planar images of the chest, abdomen and pelvis are shown. **B)** Planar anterior pelvis imaging at 5 h (left) and 24 h (right) post-injection clearly delineated the lesion. Arrows point to the tumor which was close to the bladder. Adapted from (Erb and Nabi, 2000).

After this initial report, a number of anti-CEA Fab’ fragments with various radiolabels have been used in the detection of metastatic and recurrent colorectal carcinomas (Fig. 2) (Erb and Nabi, 2000; Fuster et al. 2003; Ghesani et al. 2003; Goldenberg et al. 1997; Hladik et al. 2001; Moffat et al. 1996; Sanidas et al. 2003; Willkomm et al. 2000). To assess the performance and potential clinical impact of this Fab’ fragment-based tracer, a study in 210 presurgical patients with advanced recurrent or metastatic colorectal carcinomas was conducted (Moffat et al. 1996). Imaging with conventional diagnostic modalities, as well as surgery and histology, was also performed to validate the imaging results. It was found that the sensitivity of the Fab’ fragment-based SPECT imaging was superior to that of conventional modalities in the extrahepatic abdomen (55% vs 32%) and the pelvis (69% vs 48%) and the Fab’ fragment-based findings complemented those of conventional modalities in the liver. Among the patients with known disease, the positive predictive value was significantly higher when both modalities were positive (98%) than with each modality alone (~70%), thus potentially avoiding the need for histologic confirmation when both tests are positive. Less than 5% of the patients developed HAMA reaction to the radiolabeled anti-CEA Fab’ fragment after a single injection. Taken together, radiolabeled Fab’ fragment can afford high-quality, same day cancer detection with an inexpensive and readily available radionuclide. It can also add clinically significant information in assessing the extent and location of diseases in colorectal cancer patients, usually without inducing a HAMA response. In 1996, a ^99m^Tc-labeled anti-CEA Fab’ fragment (arcitumomab; CEA-Scan; Immunomedics, Inc.) was approved by the United States Food and Drug Administration (FDA) for colorectal cancer imaging. Subsequently, CEA-Scan has also been applied for detecting other CEA-positive cancers such as breast cancer, medullary thyroid cancer and lung cancer (Goldenberg, 1997; Goldenberg et al. 1997; Goldenberg and Nabi, 1999; Goldenberg and Wegener, 1997; Malamitsi et al. 2002; Sanidas et al. 2003).

In 2000, a study was conducted to compare the imaging efficiency of ^18^F-fluorodeoxyglucose (^18^F-FDG; t_1/2_: 109.8 min) PET, the “gold standard” of clinical cancer imaging (Gambhir et al. 2001), with CEA-Scan SPECT in colorectal cancer patients (Willkomm et al. 2000). Patients previously treated for colorectal carcinoma and were suspected for recurrence, were examined by both imaging modalities. CEA-Scan correctly detected 8 of 9 local recurrences, whereas ^18^F-FDG PET was able to detect all 9 cases with 1 case being false-positive. Liver metastases were confirmed in 9 patients by ^18^F-FDG PET but in only 1 patient by CEA-Scan SPECT. Two cases with lymph node metastases and 2 cases with lung metastases were correctly identified by ^18^F-FDG PET, none of which detected by CEA-Scan SPECT. Lastly, bone metastases were identified in 1 patient by ^18^F-FDG PET but not by CEA-Scan SPECT, whereas bone marrow infiltration was diagnosed by both imaging modalities. These results indicated that ^18^F-FDG PET and CEA-Scan SPECT are both suitable for detecting local recurrences of colorectal carcinoma. However, ^18^F-FDG PET is clearly superior in the detection of distant metastases (e.g. in the liver, bone and lung) and lymph node metastases (Libutti et al. 2001).

The absolute level of tumor uptake is typically higher for radiolabeled mAbs than the radiolabeled Fab’ fragments. However, lesions with good vascularization, vascular permeability and antigen accessibility can be detected earlier and with higher sensitivity by radiolabeled Fab’ fragments than intact mAbs, primarily due to faster background clearance despite the lower absolute tumor uptake. On the other hand, the smaller size and faster clearance of Fab’ fragments also brings some problems, most notably the prominent renal uptake. To solve this problem, cationic amino acids (such as lysine and arginine) and their derivatives have been used to reduce the renal uptake of various tracers in animals (Behr et al. 1996). Successful renal uptake reduction in patients, injected with ^99m^Tc-labeled anti-CEA Fab’ fragments, was also achieved using amino acid infusion (Behr et al. 1996). Meanwhile, many other anti-CEA antibody fragments have also been explored for better imaging results.

### Single chain Fv-based imaging

A single chain Fv (scFv; 25 kDa) fragment is consisted of the variable heavy (V_H_) and light (V_L_) regions of an antibody, linked with a short peptide sequence (Huston et al. 1988). ScFv fragments can be rapidly cleared from the blood (half-life less than a few hours), which can give very high tumor-to-blood ratio. They also have good penetration into solid tumors because they are less than onefifth the size of an intact mAb. Another advantage of scFv fragments is the reduced immunogenicity, because of the elimination of amino acid residues not involved in antigen binding.

In 1996, a bacteriophage library was used to select MFE-23, the first high-affinity scFv directed against CEA (Begent et al. 1996). After labeling with ^123^I, this scFv was tested in normal and colorectal carcinoma patients. It exhibited superior diagnostic characteristics than conventional imaging modalities, such as CT, in that it can image hepatic or abdominal metastases not detectable by any other modalities. The high tumor-to-blood ratios achieved in this clinical study explained why imaging with ^123^I-labeled MFE-23 was more sensitive than CT scans. Subsequently, the crystal structure of MFE-23 and its intermolecular contacts with CEA was elucidated (Boehm et al. 2000). In 2000, MFE-23 was labeled with ^125^I (t_1/2_: 60.1 d) for image-guided surgery (Chester et al. 2000; Mayer et al. 2000). It was given intravenously before surgery and a hand-held gamma-detecting probe was used to locate the tumor in the operative field. Thirty-four colorectal carcinoma patients (17 primary tumors, 16 liver metastases and 1 anastomotic recurrence) and 1 patient with liver metastases of pancreatic carcinoma received ^125^I-labeled MFE-23 at various time points before surgery. ^125^I-labeled MFE-23 showed good tumor localization, with an overall diagnostic accuracy of 84% when compared with histology. The short time interval between tracer injection and operation, the lack of significant toxicity and the relatively simple production in bacteria makes MFE-23 highly amenable for image-guided surgery.

Despite their small size (25 kDa), most of the radiolabeled scFvs did not perform well in pre-clinical and clinical studies because of their poor tumor retention (Shively, 2007). The fast blood clearance significantly hampered their potential in cancer diagnosis, although the high tumor-to-blood ratios would otherwise make them attractive as imaging agents. Besides the fast clearance, low avidity (due to the monovalency) of scFv fragments is another reason why they are not optimal for cancer imaging.

To improve the pharmacokinetics of scFv fragments, they were combined with antibody Fc fragments to form scFv-Fc chimeras which contain two scFv fragments (~105 kDa). The behavior of three anti-CEA scFv-Fc variants (I253A, H310A and H310A/H435Q) with differential serum persistence has been studied for tumor imaging (Kenanova et al. 2007). Biodistribution studies in CEA-positive tumor-bearing mice revealed that the ^111^In-labeled I253A fragment with the slowest blood clearance (serum half-life: 27.7 h) had the highest tumor uptake (44.6 percentage injected dose per gram of tissue [%ID/g] at 24 h p.i.), whereas the radiometal-labeled H310A/H435Q fragment with the most rapid elimination (serum half-life: 7.1 h) only had a maximum tumor uptake of 28.0%ID/g at 12 h p.i. The H310A fragment which has an intermediate serum half-life exhibited an intermediate level of tumor uptake. Biodistribution studies also showed that all three fragments were eliminated primarily through the liver and the hepatic radioactivity accumulation correlated with the rate of tracer clearance. Dosimetry estimation based on ^125^I-labeled scFv-Fc fragments suggested that ^131^I-labeled H310A/H435Q may be a promising candidate for radioimmunotherapy. Clearly, bivalent antibody fragments are superior for tumor targeting and imaging than the monovalent analogs (e.g. Fab’ and scFv) in many aspects. Besides scFv-Fc, several other forms of antibody fragments have also been explored for CEA-targeted cancer imaging.

### Diabody- and minibody-based imaging

Diabodies are the dimeric forms of scFv with a molecular weight of about 55 kDa (Carmichael et al. 2003; Li et al. 2006; Perez et al. 2006; Yazaki et al. 2001a). Radiolabeled diabodies have shown promise as in vivo tumor imaging agents in pre-clinical studies mainly due to two reasons. First, they have high avidity (bivalent) binding to the tumor antigens which gives better tumor retention than Fab’ or scFv fragments; Second, their intermediate size leads to improved tumor-to-blood ratios than intact antibodies. Because of their high retention and metabolism in the kidneys, it was proposed that radioiodine would be a preferred choice for diabody labeling since radioiodine can be rapidly excreted from the kidney once metabolized (Williams et al. 2001). However, the drawback to radioiodinated agents is that they may also be rapidly metabolized in tissues including the tumor, especially if the antigen undergoes internalization upon antibody or antibody fragment binding.

Although most anti-CEA antibodies undergo negligible internalization, higher tumor uptake and persistence was observed for radiometal-labeled (e.g. ^111^In) anti-CEA antibodies than the radioiodinated analogs (Yazaki et al. 2001b). For radiometal-labeled antibody fragments, this advantage is offset by high kidney retention, likely due to the slow excretion of the radiometal-labeled metabolites than the radioiodinated counterparts. Therefore, poly(ethylene glycol) (PEG) has been incorporated to improve the biodistribution of an anti-CEA diabody (Li et al. 2006). A number of other modifications have also been investigated for improving the in vivo kinetics of antibody fragments, such as albumin infusion (Muller et al. 2007), streptococcal protein G infusion (Stork et al. 2007), biotinylation (Barat and Wu, 2007) and humanization according to its crystal structure (Yazaki et al. 2004). When fused with luciferases, anti-CEA diabodies have been successfully used for the delineation of CEA-positive tumors with optical bioluminescence imaging (Venisnik et al. 2007; Venisnik et al. 2006).

Another type of antibody fragments, termed “minibodies”, has also been reported for CEA imaging. The chimeric T84.66 minibody is an engineered antibody construct (80 kDa) that exhibits bivalent binding and sub-nanomolar affinity to CEA (Wong et al. 2004). In animal models, the minibody demonstrated high tumor uptake, similar activity as intact antibodies with substantially faster clearance rate and superior tumor-to-blood ratios than the (Fab’)_2_ fragment, all of which makes it very attractive for further evaluation as an imaging and therapeutic agent (Yazaki et al. 2001b). A pilot clinical study was thus undertaken to evaluate the biodistribution, pharmacokinetics and immunogenicity of ^123^I-T84.66 minibody and determine whether it can target the tumor in colorectal cancer patients (Wong et al. 2004). Ten patients with biopsy-proven colorectal cancer each received 5–10 mCi (~1 mg) of the ^123^I-labeled minibody i.v. followed by serial SPECT and/or gamma camera scans, as well as blood and urine sampling over the next 3 days. Tumor detection was achieved with the ^123^I-labeled anti-CEA minibody in seven of the eight patients who did not receive neoadjuvant therapy before surgery. During surgery, no tumor was detected in one patient and only a 2-mm nodule was seen in another patient. Mean serum residence time of the radiolabeled minibody was 29.8 h (ranging between 10.9 h and 65.4 h) and no tracer-related adverse reactions were observed. All 10 patients were evaluated for immune responses to the minibody and no significant responses were found. This study showed that the T84.66 minibody was able to target colorectal tumors with a faster blood clearance than the intact mAb, however the mean residence time of the minibody in patients was significantly longer than that predicted from murine models. Further evaluation of its biodistribution and pharmacokinetic properties using a longer lived radionuclide (such as ^111^In) will be necessary in future studies.

### Pretargeted imaging

The search of the most suitable engineered antibody fragments for CEA targeting and imaging continues. Another strategy has also been explored for improving the tumor-to-background contrast: pretargeting. Typically, either the avidin/streptavidin-biotin pair or a bispecific antibody (BAb) is used (Gasparri et al. 1999; Sharkey et al. 2005). A certain waiting period is needed for the first agent to clear from the circulation and reach the tumor site. Sunsequently, a second agent is administered which will target and bind to the first agent, thus giving excellent tumor contrast if the system is designed properly.

The most common strategy for tumor pretargeting involves the use of the streptavidin-biotin recognition system (Casalini et al. 1997; Hnatowich et al. 1993; Karacay et al. 1997; Li et al. 2005). In 1993, tumor localization was investigated using a two-step pretargeting system, in which radiolabeled biotin was administered after streptavidin injection (Hnatowich et al. 1993). In the control group, nude mice bearing CEA-positive tumors (LS174T colorectal cancer) received ^111^In-labeled anti-CEA antibody or ^111^In-labeled streptavidin were sacrificed at 5 h p.i. In the pretargeting group, the animals received unlabeled streptavidin followed by ^111^In-labeled biotin at 3 h later. Because of the lower levels of labeled streptavidin in the liver and the blood, the tumor-to-normal tissue ratios were higher for ^111^In-labeled streptavidin than those of the ^111^In-labeled anti-CEA antibody, although the absolute tumor accumulation of administered radioactivity was lower for ^111^In-labeled streptavidin. When unlabeled streptavidin was administered first and followed by ^111^In-labeled biotin (pretargeting), the tumor uptake was further reduced. However, because the radioactivity concentration in the normal tissues was reduced to a even greater extent, the tumor-to-blood and tumor-to-liver ratios were 10.6 and 2.2 respectively for the pretargeting group, significantly higher than those for the ^111^In-labeled anti-CEA antibody (1.5 and 0.5 respectively). Improvement in tissue-to-muscle ratios was also seen in all tissues sampled except the kidney. It should be noted that an intact antibody circulates for days in the blood with virtually no tumor contrast at this early time point (5 h p.i.). Most likely the radiolabeled antibody would have outperformed the radiolabeled streptavidin, as well as the two-step pretargeting approach, at late time points after it has cleared from the circulation and accumulated more in the tumor.

In 1996, a three-step system for tumor pretargeting was introduced (Magnani et al. 1996). To evaluate the use of pretargeted immunoscintigraphy in the diagnosis and follow-up of patients with medullary thyroid carcinoma, twenty-five patients with histologically proven disease were enrolled (Magnani et al. 1996). A biotinylated anti-CEA mAb was injected first. One day later, avidin was administered i.v. followed by ^111^In-labeled biotin after another day. Six primary tumors, diagnosed by increased calcitonin levels, were all successfully imaged. Forty-seven suspected recurrences based on elevated blood tumor markers were detected and confirmed by cytology or histology. In one case, SPECT imaging enabled the detection of small lymph nodes with diameters of 4–7 mm. These lesions, not considered to be neoplastic based on ultrasound imaging, were confirmed to be neoplastic after fine needle aspiration. Not only did pretargeted immunoscintigraphy correctly localize primary tumors and recurrences in medullary thyroid cancer patients, false-negatives (when the tumor does not express high level of CEA therefore not detectable by radiolabeled anti-CEA antibodies) could also potentially be avoided since this three-step strategy can significantly enhance the tumor contrast, thus allowing for accurate detection of lesions with low CEA expression level.

This pretargeting strategy has also been used for radioimmunotherapy of colorectal cancer in mouse xenograft models (Karacay et al. 1997; Sharkey et al. 1997). In a recent study, the avidin-biotin system was further optimized through the use of a streptavidin conjugate of the chimeric mAb T84.66 (Jia et al. 2007). Tumor uptake of ^111^In-labeled biotin peaked at 3.3% ID/g at 15 minutes p.i. Tracer clearance from the blood and normal organs was extremely rapid with a tumor-to-blood ratio of about 20:1 at 24 h p.i., thus making it a promising strategy for the imaging of CEA-positive cancers. However, since the absolute tumor uptake is low, this method needs to be further optimized for potential therapeutic applications.

One major disadvantage of the avidin/streptavidin-biotin-based systems is that avidin/streptavidin can cause severe immune responses in certain cases (Marshall et al. 1996; Schetters 1999). Therefore, another method has been explored which uses a BAb and a radiolabeled hapten peptide (Gautherot et al. 1997; Gautherot et al. 1999; Gautherot et al. 2000; Karacay et al. 2000). The immunogenicity of BAbs can be eliminated by the use of humanized antibody fragments (Boerman et al. 2003; Chang et al. 2002). Initially, BAbs were developed as tumor targeted delivery agents for certain drugs. BAbs can be generated as small, multivalent, antigen-binding fragments with improved pharmacokinetic properties than the primary antibodies, meanwhile also capable of binding to a versatile bivalent hapten peptide which contains an imaging label (Chang et al. 2002; Rossi et al. 2005).

**Figure 3:**
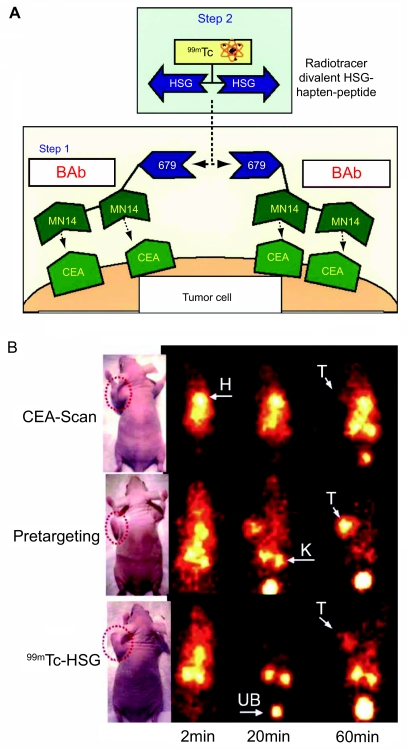
Pretargeted SPECT imaging of CEA expression. **A)** The mechanism of bispecific antibody-based pretargeting. **B)** In vivo distribution kinetics of CEA-scan (a 99mTc-labeled anti-CEA Fab’ fragment), 99mTc-labeled HSG peptide following a pretargeted bispecific antibody and 99mTc-labeled HSG peptide alone in LS174T tumor-bearing mice. T: tumor; H: heart; K: kidney; UB: urinary bladder. Adapted from (Sharkey et al. 2005).

A humanized bispecific fusion protein of the two Fv portions from an anti-CEA mAb (hMN-14; labetuzumab) and another antibody, which recognizes an inert hapten peptide (histamine-succinyl-glycine; HSG), was explored for localizing ^99m^Tc-labeled HSG to human colonic tumors in a xenograft model (Sharkey et al. 2005; Sharkey et al. 2003). The results based on this pretargeting strategy were compared to the data obtained from the clinically used CEA-Scan (Fig. 3). Tumors as small as 0.15 g were detected within 1 h of ^99m^Tc-HSG administration, with tumor-to-blood ratios significantly increasing over time (10:1 and 100:1 at 1 and 24 h p.i., respectively). Comparing with CEA-Scan, this pretargeting strategy increased the tumor uptake by ten-fold (to ~20% ID/g) under optimal conditions. Recently, it was shown that pretargeting could localize tumors in the lungs within 1.5 h p.i. of the radiolabeled HSG peptide, while ^18^F-FDG PET failed to detect the tumor (Goldenberg et al. 2008). Autoradiography demonstrated selective tumor targeting within the lungs, including metastases less than 0.3 mm in diameter. It was concluded that BAb-based pretargeting is highly specific for imaging micro-metastatic lesions and may thus provide a complementary method to ^18^F-FDG PET in the clinical setting.

The major advantage of SPECT imaging is that it can be used for simultaneous imaging of multiple radionuclides since the gamma rays emitted from different radioisotopes can be differentiated based on the energy (Berman et al. 1994). Thus, SPECT can potentially allow for simultaneous detection of multiple biological events with multiple isotopes, which is not possible with PET. However, dual-isotope SPECT imaging has not been widely used and it is unclear whether simultaneous dual-isotope imaging can offer significant advantages over single radionuclide imaging. In the past, gamma cameras and SPECT imaging systems were much more readily accessible than PET systems (Blake et al. 2003). Therefore, the number of literature reports on SPECT imaging of CEA far exceeded the number of PET studies, which will be the focus of the remaining text.

## PET Imaging of CEA Expression

PET has much higher sensitivity than SPECT, typically 10% and <0.1% respectively (Gambhir, 2002). Due to the high cost and limited availability, PET imaging was not employed for CEA imaging until the late 1990s. With the continuous developmental effort, state-of-the-art small animal PET scanners can have spatial resolution (<1 mm) comparable to SPECT and they are also becoming increasingly widely available (Chatziioannou, 2005; Stickel et al. 2007). Therefore, PET imaging of various molecular cancer markers has flourished over the last several years (Cai and Chen, 2007a; Cai and Chen, 2008; Cai et al. 2008a; Cai et al. 2008b). Similar to SPECT imaging, the PET isotopes with short half-lives are more suitable for labeling smaller molecules while the isotopes with longer half-lives are needed for antibody labeling to match its relatively long circulation half-life.

### Positron emitter labeled antibodies

Various PET isotopes have been used to label mAbs for radioimmunoPET imaging of cancer. MAbs directed against CEA was labeled with ^124^I, a positron emitter with a half-life of 4.2 days (Westera et al. 1991). Mice xenografted with CEA-positive tumors were visualized by PET imaging after injection of the ^124^I-labeled mAb. Comparison of the PET and biodistribution studies indicated that radioimmunoPET could provide more accurate radiation dosimetry estimation for radioimmunotherapy, in addition to potentially more precise diagnosis.

Other positron emitting isotopes such as ^76^Br (t_1/2_: 16.0 h) (Lovqvist et al. 1999; Lovqvist et al. 1995; Lovqvist et al. 1997a) and ^64^Cu (t_1/2_: 12.7 h) (Li et al. 2008) were also used to label anti-CEA antibodies. For the ^76^Br-labeled anti-CEA mAb, tumor sites could be readily identified by PET imaging from 46 h p.i. onwards. Interestingly, the concentration of ^76^Br in the tumor, blood and most normal tissues was higher than that of ^124^I at all time points. It was suggested that this phenomenon was mainly due to the catabolism of radiolabeled mAb which resulted in free radiohalogen: ^76^Br was persistently retained in the tumor while ^124^I was rapidly excreted. When compared with ^18^F-FDG and ^11^C (t_1/2_: 20.4 min) labeled methionine, the ^76^Br-labeled mAb was superior to both agents in subcutaneous tumor models. Further, ^76^Br-labeled mAb and ^18^F-FDG were equally successful for the identification of liver metastases, both outperformed SPECT imaging with radiolabeled anti-CEA mAbs (Lovqvist et al. 1997b).

### Positron emitter labeled antibody fragments

Antibody fragments, when designed and radiolabeled properly, are more advantageous than intact antibodies for imaging applications. An early study investigated whether the Fab’ fragment (termed “CEA-Scan” when labeled with ^99m^Tc) could be labeled with a positron-emitting nuclide: ^94m^Tc (t_1/2_: 52.5 min) (Griffiths et al. 1999). “Instant kits” containing the lyophilized Fab’ fragment of an anti-CEA IgG (arcitumomab) were reconstituted with ^94m^Tc. Radio-analyses of the ^94m^Tc-labeled Fab’ fragment indicated that ^94m^Tc could be readily incorporated in a similar fashion as ^99m^Tc-labeling. Although it was suggested that ^94m^Tc-labeling would enable the investigation of this agent for PET imaging of cancer, no further preclinical or clinical research was reported regarding its tumor targeting capability in vivo.

To the best of our knowledge, no PET imaging of tumor CEA expression with scFvs was reported due to the poor tumor targeting characteristics of this class of antibody fragments (Holliger and Hudson, 2005; Wu and Senter, 2005). To date, PET imaging of CEA is primarily based on minibodies or diabodies (Cai et al. 2007d; Griffiths et al. 2004; Sundaresan et al. 2003; Wu et al. 2000). An anti-CEA minibody was first labeled with ^64^Cu for PET imaging of cancer (Wu et al. 2000). DOTA (1,4,7,10-tetraazacyclododecane-1,4,7,10-tetraacetic acid), the most commonly used macrocyclic chelator for labeling a variety of radiometals such as ^64^Cu (Cai et al. 2006a; Cai et al. 2006c; Liu et al. 2007), was also used in this study for ^64^Cu-labeling. In vivo distribution of the tracer was evaluated in athymic mice bearing both LS174T colorectal carcinoma (CEA-positive) and C6 rat glioma (CEA-negative) tumors. Five hours after injection of the ^64^Cu-labeled anti-CEA minibody, microPET imaging showed significantly higher uptake in the CEA-positive tumor (17.9 ± 3.8% ID/g) than the control C6 tumor (6.0 ± 1.0% ID/g). Significant radioactivity uptake was also seen in liver which is likely attributed to two major factors: the reticuloendothelial system uptake of the minibody due to its relatively large size (80 kDa) and the possible trans-chelation of ^64^Cu (Cai et al. 2007b; Cai and Chen, 2007b; Cai and Chen, 2008).

^124^I- or ^64^Cu-labeled anti-CEA diabody was initially evaluated in mice xenografted with LS174T tumors (Olafsen et al. 2004a; Sundaresan et al. 2003). For the ^124^I-labeled diabody, PET imaging showed specific localization to CEA-positive tumors and low activity elsewhere in the mice (Sundaresan et al. 2003). Tumor-to-background ratios were 4.0 and 10.9 at 4 and 18 h, respectively. At 18 h p.i., the radioactivity in the normal organs (including the liver and kidneys) were nearly fully cleared, leaving only the CEA-positive tumors with excellent contrast. Tumors as small as 11 mg (<3 mm in diameter) could be imaged by the ^124^I-labeled anti-CEA diabody, which significantly out-performed ^18^F-FDG PET. For the ^64^Cu-labeled anti-CEA diabody, the tumor-to-background ratio was 4.6 at 18 h p.i. (Olafsen et al. 2004b), significantly lower than the ^124^I-labeled version. Further, possible trans-chelation of ^64^Cu also resulted in elevated liver activity (19.4%ID/g at 4 h p.i.).

^124^I and ^64^Cu each has several disadvantages for PET imaging applications. For example, both isotopes have low positron efficiency (<25%) which resulted in low signal intensity. The relatively long half-lives of the two isotopes may not be optimal/necessary for the labeling of diabodies since diabodies typically clear quite rapidly from the circulation. For clinical translation, ^18^F-labeled diabody is potentially more suitable since ^18^F offers the advantages of broad availability, a high positron yield (nearly 100%) and a short half-life ideal for routine clinical use.

An imaging figure of merit (IFOM) analysis, a measure of how rapidly a statistically significant tumor image can be acquired (Williams et al. 2001), was performed for combinations of different radionuclides across various engineered antibody fragments (scFv, diabody, minibody, F(ab’)_2_ and intact antibody) based on murine biodistribution data. This analysis predicted that diabody should provide the best vehicle for ^18^F and optimal imaging should occur at 1–2 h after injection (Williams et al. 2001).

**Figure 4:**
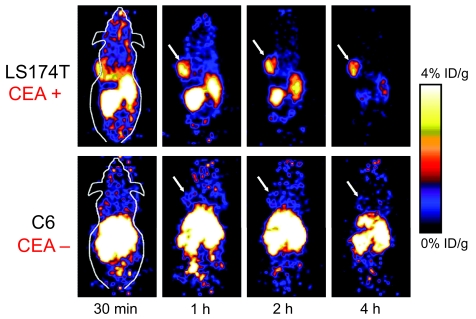
Dynamic small-animal PET scans of LS174T human colorectal (CEA-positive) tumor-bearing mice and C6 rat glioma (CEA-negative) tumor-bearing mice after inejction of a 18F-labeled anti-CEA diabody. Coronal whole-body slices that contained the tumors (arrows) are shown. Adapted from (Cai et al. 2007d).

Such prediction was confirmed by a recent study (Cai et al. 2007d). An anti-CEA diabody was labeled with ^18^F which gave high-contrast PET images of tumors, with tumor-to-normal tissue ratios as high as 6.2 at 4 h p.i. (Fig. 4). Most importantly, the tumor contrast was sufficiently high to allow for PET imaging as early as 1 h p.i. The choice of ^18^F as the best PET radionuclide for diabodies meets the needs and experience of PET clinicians who are familiar with the equipment used for and the interpretation of conventional ^18^F-FDG PET scans (Shively, 2007). It is obvious that imaging agents that require a patient to stay overnight or to return on the following day dramatically increase the cost of the procedure and patient inconvenience. Because of the slow clearance of intact antibodies, antibody-based PET or SPECT scans often require the scans be performed at up to 3 days after tracer injection. This proof-of-principle study clearly suggested the enormous potential of ^18^F-labeled diabodies for radioimmunoPET imaging in the clinic. Upon further improvement in the immunoreactivity and radiolabeling yield, ^18^F and diabody may prove to be the optimal combination for clinical diagnosis of cancer.

### Pretargeted PET imaging

BAb-based pretargeting strategy has been applied for PET imaging. A peptide, DOTA-D-Tyr-D-Lys(HSG)-D-Glu-D-Lys(HSG)-NH_2_, was synthesized and labeled with ^124^I (McBride et al. 2006). Pretargeted imaging with the ^124^I-labeled peptide was tested in nude mice bearing LS174T tumors that were first injected with a BAb that recognizes both CEA and HSG. Comparisons were performed between animals injected with a ^124^I-labeled anti-CEA Fab’ fragment, ^18^F-FDG, the same peptide labeled with ^111^In and pretargeted with the BAb, or the ^124^I-labeled peptide alone. It was found that the ^124^I-labeled peptide cleared quickly from the blood with no evidence of tumor targeting. When pretargeted with the BAb, tumor uptake of the tracer increased by 70-fold. The efficient and rapid clearance of the tracer from normal tissues enabled clear visualization of the tumor within 1–2 h p.i. Tumor uptake measured at necropsy was found to be 3- to 15-fold higher than that of the ^124^I-labeled Fab’ fragment and the tumor-to-blood ratios were 10- to 20-fold higher. Tumor visualization with ^18^F-FDG PET at approximately 1.5 h p.i. was also quite satisfactory. However, the substantially higher uptake of ^18^F-FDG in several normal tissues made image interpretation in the pretargeted animals far less ambiguous than with ^18^F-FDG PET.

Subsequently, it was reported that this BAb-based pretargeting approach not only significantly enhanced tumor-to-normal tissue ratios but also provided higher signal intensity in the tumor (Sharkey et al. 2007). With anti-CEA BAb-based pretargeting, it was possible to visualize micro-metastases of colonic cancer as small as 0.1 to 0.2 mm in diameter, whereas ^18^F-FDG PET failed to localize these lesions in a nude mouse model. ^68^Ga (t_1/2_: 68 min), a PET isotope that can be produced by generators, has also been reported for pretargeted imaging of CEA expression in tumor models (Griffiths et al. 2004).

## Summary

A wide variety of strategies have been explored for CEA-targeted cancer imaging, including the use of intact antibodies, a number of differently sized antibody fragments and pretargeting strategies (Table 1). For antibody- and antibody fragment-based imaging, it appears that intermediate sized fragments are the optimal choices for imaging applications. In particular, diabodies are very promising agents since they can retain the high avidity of the original intact antibody while at the same time exhibiting short circulation half-lives. BAb-based pretargeting method can be useful for both CEA imaging and CEA-targeted immunotherapy. For diagnostic purposes, BAb-based pretargeted imaging was reported to be capable of outperforming ^18^F-FDG PET, the current “gold standard” for clinical cancer diagnosis and staging. However, pretargeting strategy still has a long way to go before it can be widely used for therapeutic applications. Although it can give excellent tumor contrast in many cases, the absolute uptake level in the tumor is still far less that that of radiolabeled antibodies. Therefore, radiolabeled intact antibodies are still the best choices for radioimmunotherapy of CEA-positive tumors.

Translating novel anti-cancer agents from bench to bedside is typically time-consuming and quite expensive (DiMasi et al. 2003). Multiple steps in preclinical development, especially the investigational new drug (IND)-directed toxicology, significantly slowed down this process (Cai and Chen, 2006; Cai et al. 2006b). Development of protein-based therapeutics, such as those targeting CEA, is even more difficult and expensive comparing to traditional small molecule-based drugs. Not only are proteins relatively difficult to manufacture in large quantities, good laboratory/manufacturing practice (GLP/GMP) compliance is also much more technically challenging. Currently, a vast number of new anti-cancer therapies are in preclinical and clinical testing. Whether antibody- and/or antibody fragment-based CEA imaging and/or therapy is cost effective and whether it can be included in the clinical regimens for various cancers is still debatable. Development of peptide-based tracers should be explored for CEA imaging in the future.

A significant portion of the preclinical CEA imaging studies used the LS174T colorectal cancer xenograft model, which has very high level of CEA expression. They may not truly reflect the clinical situation where CEA expression is quite heterogenous, not only between patients but also between the different lesions in the same patients. Studies in other more clinical relevant orthotopic or transgenic mouse models will more closely mimic the clinical situation, thereby providing more clinically relevant findings. Another aspect not well studied is the quantitative imaging of CEA expression in vivo. Although it is generally assumed that noninvasive imaging results correlate with the target expression level, such assumption has not been extensively validated. In most reports, two tumor models are used where one acts as a positive control and the other as a negative control. Quantitative correlation between the target expression level in vivo and the noninvasive PET imaging data is rare (Cai et al. 2007a; Cai et al. 2007c; Zhang et al. 2006). Such correlation is critical for future treatment monitoring applications, as it would be ideal to be able to monitor the changes in the target expression level quantitatively, rather than qualitatively, in each individual patient.

Interestingly, a significant portion of the clinical data regarding CEA imaging comes from early studies more than 10 years ago, although at that time the term “molecular imaging” has not been widely spread across the scientific community. The explosion of molecular imaging research over the last decade is partially, if not mostly, due to the increasingly wider availability of scanners dedicated to small animal studies. However, with an overwhelming number of animal studies reported every week, clinical translation has become more and more difficult in the United States due to many regulatory constraints. Many scientific societies have recently tried to work with the regulatory agencies (most notably the FDA) in order to alleviate the prohibitively expensive requirements before initial clinical evaluation of molecular imaging agents, especially PET/SPECT agents since they are administered at doses many orders of magnitude below the pharmacologically active level. To date, most of the imaging probe development comes from academic institutions. Significant involvement from clinicians, pharmaceutical industries, government agencies are needed to allow for rapid first-in-human evaluations and subsequent clinical trials. The molecular imaging field has grown extremely fast over the last decade and the value of molecular imaging in drug development and cancer patient management is getting more widely accepted (Rudin and Weissleder 2003; Willmann et al. 2008). It is expected that in the foreseeable future molecular imaging will be routinely applied in clinical trials and cancer patient management.

## Figures and Tables

**Table 1:** Extensive investigation has been carried out for CEA-targeted cancer imaging.

Fragment	Isotopes	Stage	Major advantages	Major disadvantages	Selected ref.
Intact antibody	^131^I, ^111^In, ^99m^Tc, ^64^Cu, ^124^I, ^76^Br	clinical	high affinity, good therapeutic potential	prolonged circulation, low contrast, high cost	(Li et al. 2008; Patt et al. 1990)
Fab’	^99m^Tc, ^123^I, ^94m^Tc	clinical	fast blood clearance	prominent renal uptake, low avidity	(Ghesani et al. 2003; Griffiths et al. 1999)
ScFv	^123^I, ^125^I, ^111^In	clinical intra-operative	fast blood clearance, low immunogenicity	fast blood clearance, low tumor uptake	(Begent et al. 1996; Mayer et al. 2000)
ScFv-Fc	^125^I, ^111^In	preclinical	good tumor contrast, good therapeutic potential	slow clearance, high liver uptake	(Kenanova et al. 2007)
Diabody	^64^Cu, ^124^I, ^18^F	preclinical	high affinity, high tumor contrast, suitable clearance rate	high kidney uptake	(Cai et al. 2007d; Olafsen et al. 2004b)
Minibody	^123^I, ^64^Cu	clinical pilot	high affinity, high tumor contrast, suitable clearance rate	pharmacokinetic complexity	(Wong et al. 2004; Wu et al. 2000)
Pretargeting	^111^In, ^99m^Tc, ^124^I, ^68^Ga	mainly preclinical	excellent tumor contrast, high sensitivity	complicated procedure, not suitable for therapy	(McBride et al. 2006; Sharkey et al. 2005)
